# PARP1′s Involvement in RNA Polymerase II Elongation: Pausing and Releasing Regulation through the Integrator and Super Elongation Complex

**DOI:** 10.3390/cells11203202

**Published:** 2022-10-12

**Authors:** Elena A. Matveeva, Hejer Dhahri, Yvonne Fondufe-Mittendorf

**Affiliations:** 1 Department of Molecular and Cellular Biochemistry, University of Kentucky, Lexington, KY 40536, USA; 2 Department of Epigenetics, Van Andel Institute, Grand Rapids, MI 49503, USA

**Keywords:** transcription elongation, alternative splicing, pause–release, chromatin, epigenetics

## Abstract

RNA polymerase elongation along the gene body is tightly regulated to ensure proper transcription and alternative splicing events. Understanding the mechanism and factors critical in regulating the rate of RNA polymerase II elongation and processivity is clearly important. Recently we showed that PARP1, a well-known DNA repair protein, when bound to chromatin, regulates RNA polymerase II elongation. However, the mechanism by which it does so is not known. In the current study, we aimed to tease out how PARP1 regulates RNAPII elongation. We show, both in vivo and in vitro, that PARP1 binds directly to the Integrator subunit 3 (IntS3), a member of the elongation Integrator complex. The association between the two proteins is mediated via the C-terminal domain of PARP1 to the C-terminal domain of IntS3. Interestingly, the occupancy of IntS3 along two PARP1 target genes mimicked that of PARP1, suggesting a role in its recruitment/assembly of elongation factors. Indeed, the knockdown of PARP1 resulted in differential chromatin association and gene occupancy of IntS3 and other key elongation factors. Most of these PARP1-mediated effects were due to the physical presence of PARP1 rather than its PARylation activity. These studies argue that PARP1 controls the progressive RNAPII elongation complexes. In summary, we present a platform to begin to decipher PARP1′s role in recruiting/scaffolding elongation factors along the gene body regions during RNA polymerase II elongation and gene regulation.

## 1. Introduction

Alternative splicing results in variant transcripts, with consequences in increased protein diversity and function. Of all human genes, 90–95% undergo critical alternative splicing [1] in response to normal growth and environmental signals [2,3]. Previously, it was thought that regulation of alternative splicing was pre-mRNA-encoded by transcription factors (enhancers or silencers) that bind to the pre-mRNA [4,5]. However, recent data indicate that this pre-mRNA-encoded information is inadequate to explain the ability of splicing factors to recognize weak splice sites within a sea of introns [6,7,8]. This led to the co-transcriptional splicing hypothesis, whereby chromatin structure regulates not only the levels of the transcripts made but how these transcripts are spliced [9,10]. 

Two non-mutually exclusive models have been hypothesized to explain how chromatin structure regulates alternative splicing. In the kinetic model, the local chromatin structure regulates the speed by which RNA polymerase II (RNAPII) moves along the gene body [4]. Epigenetic features such as the deposition of DNA methylation [11,12,13] and histone modifications [14,15,16] may increase the nucleosome occupancy at exon/intron boundaries to effect changes in the RNAPII transcriptional rate [17,18,19,20]. On the other hand, RNA-binding proteins associating with chromatin may stall RNAPII elongation, triggering the formation of a dense heterochromatin structure [21]. In the adaptor/recruitment model, the recruitment of splicing factors by chromatin or chromatin-associated factors bridges chromatin to nascent mRNA to impact splicing decisions [22,23,24]. Based on these models, we hypothesized that factors that impact chromatin structure should play a role in splicing decisions. One such factor is poly (ADP–ribose) polymerase 1 (PARP1), which modulates the chromatin structure. 

PARP1 is recognized for its role in DNA repair and genome integrity [25,26], yet its role in splicing regulation is limited. Earlier studies showed that PARP1 indirectly regulates splicing decisions by PARylating and activating splicing factors [27,28]. We showed recently that PARP1–chromatin binding directly impacts splicing decisions, serving as a gene regulatory hub to facilitate co-transcriptional splicing [29]. We also showed that PARP1 could act in both proposed non-mutually exclusive models above. In support of the adaptor/recruitment model, PARP1 binds both chromatin and RNA [29,30] and recruits splicing factor 3B1 (SF3B1), a U2 spliceosomal member [29], to chromatin, bringing the spliceosomal complex to RNA. In support of the kinetic model, a PARP1-mediated chromatin structure creates a barrier that slows down the rate of RNAPII elongation within genes [31]. These results are consistent with previous studies showing that PARP1 creates a chromatin structure that pauses RNAPII at the promoters of heat-shock genes [32]. Nevertheless, it is still not clear how the PARP1–chromatin structure regulates RNAPII elongation. 

We theorize that PARP1-bound chromatin at exon–intron boundaries could impact RNAPII elongation by regulating the association/recruitment of RNAPII-associated elongation factors. This idea is not far-fetched, as PARP1 has been shown to control the recruitment of key factors in other cellular processes, such as in DNA repair [33] and in splicing [29]. We therefore asked whether PARP1 impacts RNAPII elongation through the recruitment of elongation factors along the gene body, with consequences in alternative splicing. RNAPII elongation begins at the promoter, with the sequential recruitment of the DSIF-NELF (DRB-sensitivity-inducing factor and negative elongation factor), followed by the Integrator complex. Immediately after RNAPII elongation starts, it pauses downstream of the transcription start site. This paused RNAPII remains associated with nascent RNA and is fully capable of resuming elongation; however, further signals are required for it to transition to productive elongation. These signals prompt the binding of the positive elongation factor (P-TEFb), followed by the Super Elongation Complex (SEC) [34,35,36], and together they form the central elongation scaffold [37]. While PARP1′s role in pause–release of RNAPII at the promoters is well recognized (through PARylation of NELF), very little is known about its role in the gene body. We therefore carried out a comprehensive study mapping the impact of PARP1 on the recruitment of elongation factors. We asked whether PARP1 aids in recruitment and/or assembly of members of the SEC and/or the Integrator complex. We show that PARP1 directly binds IntS3, a member of the Integrator complex. We also show that its association is neither dependent on PARP1′s activity nor the presence of nucleic acids. Additionally, we show that PARP1 knockdown and PARylation inhibition impact the chromatin association of elongation factors as well as their occupancy along the gene body. These studies, therefore, support the idea that PARP1 could be acting in the recruitment/assembly of elongation factors critical for RNAPII pause–release and elongation.

## 2. Materials and Methods

### 2.1. S2 Cell Culture and siRNA Mediated Knockdown

S2 Drosophila melanogaster cells were obtained from ThermoFisher Scientific, Waltham, MA, USA. Cells were grown at 25 °C in Schneider’s Drosophila medium (Life Technologies, Austin, TX, USA) to which 10% heat-activated fetal bovine serum (Sigma, St Louis, MO, USA), 100 U/mL penicillin, and 100 μg/mL streptomycin (ThermoFischer Scientific, Waltham, MA, USA) was added. The same growth conditions were maintained between controls and experimental samples. PARP1 was knocked down using siRNA, as we have shown previously [29]. To quantify the efficiency of PARP1 knockdown, we used a serial dilution of the total protein as shown in Figure S1. This method has been used by others as well [32,38]. We confirmed PARP1 depletion at the transcript level using quantitative PCR with primers 1–4 (Table S1) and protein levels using Western blot analyses.

### 2.2. S2 Cell PARP1 Inhibitor Treatment

To inhibit PARP1 PARylation, Drosophila melanogaster S2 cells were treated with 10 μM PJ34, (Sigma, St. Louis, MO, USA) a PARP1 inhibitor for 16 h. There are several inhibitors of PARP1. For instance, veliparib and niraparib selectively inhibit PARP1 and PARP2; olaparib and rucaparib, though more potent inhibitors, are less selective. We therefore chose to use PJ34, not only because it is a broad PARP inhibitor [39], but because we had used in our previous studies on PARP1’s role in transcription [29,31].

### 2.3. S2 Cell NAD+ Treatment and Heat Shock

Cells were cultured in the presence of 0.5 mM NAD+ (Sigma, St Louis, MO, USA), overnight and collected by centrifugation the next morning. To achieve the heat-shock condition, cells were incubated in a water bath for 15 min at 37 °C.

### 2.4. Expression and Purification of Recombinant Proteins

The full-length PARP1 and individual or groups of PARP1 domain plasmids were a generous gift from Dr. J. M. Pascal. Protein expressions and purification were performed according to Langelier et al., 2011 [40].The full-length IntS3 and N- and C-terminal IntS3 plasmids (GST-tagged) were a generous gift from Dr Y. Wu. Protein expressions and purification were performed according to Vidhyasagar et al., 2018 [41].

### 2.5. Recombinant PARP1 PARylation

A 1 µM quantity of recombinant PARP1 and 1 µM DNA (5′–CGT ACG CGG GTT TAA ACG A –3′) or RNA (5′–CGU ACG CGG GUU UAA ACG AG–3′) were mixed to a final volume of 20 µL in 50 mM Tris (pH 8), 50 mM NaCl, 10 mM MgCl2, and 1 mM DTT. For PARylation, 20 µL of NAD+ stock (1 mM) was added (making the final NAD+ concentration 0.5 mM) and allowed to incubate for 10 min at room temperature. The reaction was quenched with 5xLaemmli buffer, boiled for 3 min, and analyzed by SDS-PAGE and Western blot.

### 2.6. Western Blot Analyses

Western blot analyses were performed per standard protocol and input dilutions were used as a quantitative indication of signal linearity. Protein samples were resuspended in SDS loading buffer and electrophoresed on a 10% or 7.5% SDS-PAGE gel, blotted to a PVDF membrane (Thermo Scientific, Rockford, IL, USA) and sequentially probed with primary antibodies. Western-blot-based detection was performed using alkaline phosphatase-coupled secondary antibodies (Sigma, St. Louis, MO, USA) with Amersham ECF substrate for visualization (GE Healthcare, Waukesha, WI, USA) and images were obtained using Typhoon FLA 9500 (GE Healthcare, Piscataway, NJ, USA). Image Quant^TM^ TL software, Waukesha, WI, USA was used to quantify protein signals. 

### 2.7. Analyses and Quantitation of PARP1 Depletion by qRTPCR

Total RNA was purified from both WT. and PARP1 KD cells using the RNase mini kit (QIAGEN GmbH, Hilden, Germany) per the manufacturer’s instructions. cDNAs from these RNAs were then synthesized using the SuperScript™ III Reverse Transcriptase (Invitrogen, Carlsbad, CA, USA). PARP1 expression was determined by PCR with PARP1-specific primers as described previously [29,31,42]. GAPDH primer was used as housekeeping gene control. Quantitative PCR (qRTPCR) analysis was performed on a CFX96 Real-Time System (Bio-Rad, Hercules, CA, USA) machine. Reactions occurred in a final volume of 25 μL and contained Taq DNA polymerase (MB042-EUT-10000, Syd Labs, Natick, MA, USA) and Evergreen dye (Biotium, Fremont, CA, USA). Cycling parameters were as follows: 4 min at 94 °C, followed by 40 cycles of 45 s at 94 °C, 30 s at 60 °C, and 60 s at 72 °C. Melting curves were acquired (10 s at 95 °C and 60 s at 60 °C) for all samples and used to control for correct species generation. All experiments were performed three times with at least three biological replicates. See Table S1 for the primer sequences used in study.

### 2.8. Antibodies

Western blot analysis: The following primary antibodies were used: PARP1 C terminal, rabbit polyclonal (#39561, Active Motif, Carlsbad, CA, USA); Actin, mouse monoclonal (MA1-744, Thermo Fisher Scientific, Waltham, MA); H5 (Ser2P), mouse monoclonal (ab24758, Abcam, Cambridge, MA, USA); IntS3, rabbit polyclonal (PA5-72344, Thermo Fisher Scientific, Waltham, MA, USA); AF9, rabbit polyclonal (PA5-13174, Thermo Fisher Scientific, Waltham, MA, USA), Cyclin T1, rabbit polyclonal (PA5-24163, Thermo Fisher Scientific, Waltham, MA, USA); PAR rabbit polyclonal (4336-BPC-100, Trevigen, Gaithersburg, MD, USA). Secondary antibodies used: anti-rabbit (A3687) and anti-mouse (A3562) IgG (whole molecule) alkaline phosphatase antibody (Sigma).

Co-immunoprecipitation and chromatin immunoprecipitation (ChIP): PARP1, rabbit polyclonal (#39561, Active Motif, Carlsbad, CA, USA); H5 (Ser2P), mouse monoclonal (ab24758, Abcam, Cambridge, MA, USA); 4H8, mouse monoclonal (ab5408, Abcam, Cambridge, MA, USA); 8WG16, mouse monoclonal (ab817, Abcam, Cambridge, MA, USA); IntS3, rabbit polyclonal (PA5-72344, Thermo Fisher Scientific, Waltham, MA, USA); AF9, rabbit polyclonal (PA5-13174, Thermo Fisher Scientific, Waltham, MA, USA). 

### 2.9. S2 Cell Lysate Preparation

A total of 10^7^ Drosophila melanogaster S2 cells were pelleted and resuspended in 0.5 mL of RIPA buffer, to which protease inhibitors were added: 1 mM PMSF (phenylmethylsulfonyl fluoride, Sigma, St Louis, MO, USA, #10837091001), 1× protease inhibitor cocktail (EpiGentek, Farmingdale, NY, USA, #R-1101), 10 mM 3-MBZ (3-Methoxybenzylamine, Sigma, #159891), and 0.5 mM BZA (Benzylamine, Sigma, #185701). Cell suspension was put on ice for 30 min, followed by 6 cycles of sonication (each cycle: 10 s on/10 s off) on a Bioruptor300 (Diagenode, Denville, NJ, USA). The cell lysate was cleared by spinning at 13,000 rpm for 10 min. The resultant supernatant was then used for co-immunoprecipitation and Western blot analyses. 

### 2.10. Co-Immunoprecipitation and Pull-Down Assays

For the co-immunoprecipitation of PARP1 binding to proteins from S2 cells, 50 μg of protein lysate was precleared and subjected to immunoprecipitation for 2 h at 4 °C with either PARP1 or IntS3 antibodies plus Protein G Magnetic beads (ThermoFisher Scientific, Waltham, MA, USA). After washing with RIPA buffer, beads were boiled for 2 min in SDS-loading buffer and subjected to SDS-PAGE and Western blot. 

For in vitro binding experiments, PARP1 or IntS3 antibodies were used to immunoprecipitate recombinant PARP1 (full-length or its truncated mutants) bound to recombinant IntS3 (full-length, N-terminal or C-terminal) or vice versa. Protein G/Protein A magnetic beads were then added and incubated for 2 h at 4 °C. Beads were then washed several times in 1XPBS and then boiled for 2 min in SDS-PAGE loading buffer. Samples were then loaded onto SDS-PAGE and subjected to Western blot analysis. 

### 2.11. Chromatin Immunoprecipitation (ChIP) 

We performed cross-link chromatin immunoprecipitation (X-ChIP) as described by Abcam, with some modifications. A total of 1 × 10^7^ cells were resuspended in PBS and fixed with 1% formaldehyde for 10 min. Following this, the cell pellet was washed 3 times with cold PBS and again spun down at 1200 rpm for 5 min to collect the cell pellet. We then resuspended the cell pellet in a lysis buffer (50 mM HEPES-KOH (pH 7.5), 140 mM NaCl, 1 mM EDTA (pH 8), 1% SDS, 1% Triton X-100, 0.1% sodium deoxycholate, protease inhibitors) and incubated for 10 min on ice. Afterwards, the lysate was sonicated for 20 min (30 s on/30 s off) using a Bioruptor 300, (Diagenode, Sparta, NJ, USA), shearing the DNA to an average fragment size of about 150–700 base pairs. The resulting cell debris was pelleted, and the resultant supernatant then used for immunoprecipitation (IP). For each IP, 25 μg of chromatin was used. For ChIP, a lysate containing chromatin was diluted 1:10 in 1× RIPA buffer (50 mM Tris–HCl, (pH 8), 150 mM NaCl, 2 mM EDTA (pH 8), 1% NP-40, 0.1% SDS, 0.5% sodium deoxycholate, protease inhibitors). A 50 μL aliquot of chromatin was removed to serve as the input sample. Primary antibodies (PARP1, S2P, 4H8, 8WG16, IntS3, or AF9) were added to samples (10 μg per 25 μg DNA) and rotated at 4 °C for 1 h. For negative or non-specific background control, Rabbit IgG was used. 

To the antibody–chromatin complexes, Protein A/G Dynabeads (Thermo Fisher Scientific, Waltham, MA) were added and incubated overnight at 4 °C with rotation in the presence of BSA (0.2 mg/mL). Beads were then washed twice with low salt buffer (0.1% SDS, 1% Triton X-100, 2 mM EDTA, 20 mM Tris-HCl pH 8, 150 mM NaCl), followed two washes with a high salt buffer (0.1% SDS, 1% Triton X-100, 2 mM EDTA, 20 mM Tris-HCl pH 8, 500 mM NaCl). After this wash, samples were washed twice with a LiCl buffer (0.25 M LiCl, 1% NP-40, 1% sodium deoxycholate, 1 mM EDTA, 10 mM Tris-HCl, pH 8). Lastly, the specific DNA–protein complexes were eluted with 120 μL of elution buffer (1% SDS, 10 mM NaHCO3) for 15 min at 30 °C. We next incubated both the immunoprecipitated and control chromatin input samples at 65 °C overnight, to reverse cross-link DNA–protein interactions. Finally, the DNA was purified using the QIAquick PCR Purification Kit (Qiagen, Gaithersburg, MD, USA) as per the manufacturer’s protocol. Quantitative real-time PCR with primers 5–23 (Table S1) was used to quantify the amount of specific DNA fragments recovered from the immunoprecipitated DNA. These primers were designed using Integrated DNA Technologies Primer Tools. Real-time quantitative PCR (RT-qPCR) analysis was performed on a CFX96 Real-Time System (Bio-Rad). In the 25 μL PCR reactions, DNA was amplified using Taq DNA polymerase (MB042-EUT-10000, Syd Labs, Natick, MA, USA) and Eva Green dye (Biotium). The cycling parameters were as follows: 4 min at 94 °C, followed by 40 cycles of 45 s at 94 °C, 30 s at 60 °C and 60 s at 72 °C. For qPCR analysis, all values were normalized to ChIP input inside of each exon and to Exon 1 to see the distribution along the gene. Intergenic primers were used for background control (Primers 24–25, Table S1).

### 2.12. Salt Fractionation of Chromatin 

Salt fractionation of chromatin was done according to Treves et al. (54), with some modifications. Cells were harvested (2 × 10^7^) and washed twice with ice-cold phosphate-buffered saline (PBS) and centrifuged at 2000 rpm for 5 min. Pellets were resuspended in 1 mL TM2 buffer (10 mM Tris-HCl, pH 7.4, 2 mM MgCl2, 0.5 mM PMSF, 1× protease inhibitor cocktail (PIC), 0.5 mM BZA and 10 mM 3-methoxybenzylamine (3-MBZ)) for 5 min on ice with intermittent vortexing. A 60 μL quantity of 10% NP-40 was added to each sample while gently vortexing, followed by incubation on ice for 5 min with intermittent vortexing. Cells were centrifuged at 200 rpm for 5 min and washed twice with 1 mL TM2 buffer (letting sit for 10 min room temperature (RT) and centrifuged at 2000 rpm for 10 min each time). 

Pellets were resuspended in 400 μL MNase buffer with all four inhibitors: PMSF, PIC, BZA and 3-MBZ, and 5 μL of MNase; 50 U/μL was added to each sample followed by 20 min incubation at RT. A 20 μL quantity of 0.2M EGTA was added (until a final concentration of 8 mM). An 80 μL quantity was taken for Western blot (WB) sample and 50 μL for a DNA sample. The rest was centrifuged at 2000 rpm for 10 min at 4°C. A total of 80 μL of supernatant was taken for WB analysis; the remaining supernatant (~160 μL) was subjected to low-salt extraction by resuspending in 200 μL of 80 mM Triton Buffer (70 mM NaCl, 10 mM Tris-HCl, pH 7.4, 2 mM MgCl2, 2 mM EGTA, 0.1% Triton X-100, and 0.5 mM PMSF) on a rotator at 4 °C for 30 min for DNA purification and pellets. 

The pellets were centrifuged at 2000 rpm for 10 min at 4 °C. The collected supernatant was centrifuged at 13,000 rpm for 2 min, and split half and half for WB and DNA analyses (100 μL each). The pellets were subjected to mid-salt extraction by resuspending in 200 μL of 150 mM Triton Buffer (140 mM NaCl, 10 mM Tris-HCl, pH 7.4, 2 mM MgCl2, 2 mM EGTA, 0.1% Triton X-100, and 0.5 mM PMSF) on a rotator at 4 °C for 30 min. 

The pellets were centrifuged at 2000 rpm for 10 min at 4 °C. The collected supernatant was centrifuged at 13,000 rpm for 2 min, and split half and half for WB and DNA analyses (100 μL each). The pellets were subjected to high-salt extraction by resuspending in 200 μL of 600 mM Triton Buffer (590 mM NaCl, 10 mM Tris-HCl, pH 7.4, 2 mM MgCl2, 2 mM EGTA, 0.1% Triton X-100, and 0.5 mM PMSF) on a rotator at 4 °C for 30 min. 

The pellets were centrifuged at 2000 rpm for 10 min at 4 °C. The collected supernatant was centrifuged at 13,000 rpm for 2 min, and split half and half for WB and DNA analyses (100 μL each). Leftover pellets were resuspended at 100 μL of TNE buffer (10 mM Tris-HCl, pH 7.4, 200 mM NaCl, and 1 mM EDTA) and split half and half for WB and DNA analyses (50 μL each). A 20 μL quantity of each WB fraction were used to run on 10% SDS-PAGE gel. DNA samples were purified as described at QIAquick PCR Purification Kit Protocol, eluted with 50 μL of elution buffer (10 mM Tris-HCl, pH 8.5) and run on 4% sieve agarose gel (15 μL of sample with 5 μL of orange dye).

We used salt fractionation in contrast to microscopic analyses, as this gives a better method to tease PARP1’s role in the functional association of these elongation factors within the different chromatin types (heterochromatin and euchromatin). In addition, salt fractionation also presents samples to use in subsequent biochemical analysis such as Western blotting and immunoprecipitation [42]. 

### 2.13. Genes of Interest

AKAP200 (Flybase ID: FBgn0027932, symbol: CG13388)

CAPER (Flybase ID: FBgn0031883, symbol: CG11266)

## 3. Results

### 3.1. PARP1 Associates Directly with IntS3 of the Integrator Complex

We previously showed that PARP1 plays a role in RNA splicing [29], possibly by impacting RNAPII elongation along the gene body [31]. We also showed that IntS3 is part of the PARP1 interactome in S2 *Drosophila* cells [29]. Indeed, DNase I treatment of the PARP1 interacting complex did not disrupt this association, suggesting that DNA does not mediate the association of PARP1 and IntS3. Yet our studies did not provide details of this interaction. To begin these studies, we asked whether the association between PARP1 and IntS3 occurs directly, using in vitro and in vivo immunoprecipitation assays. 

#### PARP1 and IntS3 Associate In Vivo

**Figure 1:**
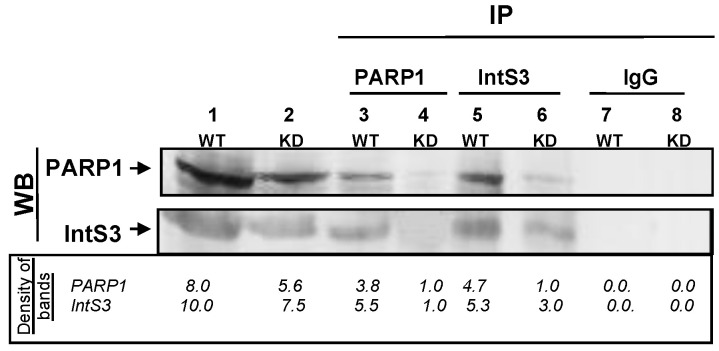
PARP1 and IntS3 associate in vivo in drosophila S2 cells. In WT cells (Lane 2), immunoprecipitation of PARP1 pulled down IntS3 as a direct interactor (Lane 3). In reciprocal experiments, immunoprecipitation of IntS3 pulled down PARP1 as an interactor (Lane 5). Knockdown of PARP1 (Figure S1) reduces the PARP1–IntS3 complex (Lane 4), but not IntS3 levels. Therefore, when the very little PARP1 caused by knockdown is bound to the beads, little IntS3 is pulled down in a corresponding level. On the other hand, since knockdown of PARP1 does not affect IntS3 levels, the higher level of IntS3 bound to the beads pulled down whatever PARP1 remained in the knockdown conditions (Lane 6). No immunoprecipitation was seen from IgG controls for either WT or KD cells (Lanes 7 and 8, respectively). Image Quant TL software was used to quantify band density (background subtracted), as shown below the Western blot image.

In the first approach, S2 nuclear lysates were subjected to immunoprecipitation using antibodies against PARP1 and IntS3 (see Materials and Methods) under highly stringent conditions (0.4 M KCl, 0.05% NP-40). First, we immobilized the PARP1 antibody onto protein G beads and performed co-immunoprecipitation assays. Western blot analyses of the resultant immunoprecipitants show that IntS3 co-immunoprecipitated with PARP1 (Figure 1, Lane 3). In a reciprocal assay, we immobilized the IntS3 antibody onto protein G beads, and immunoprecipitated IntS3 associating proteins. Subsequent Western blot analysis confirmed the co-immunoprecipitation of PARP1 with IntS3 (Figure 1, Lane 5). This interaction between PARP1 and IntS3 is specific, as control antibodies (IgG) did not immunoprecipitate either IntS3 or PARP1 (Figure 1, Lanes 7 and 8). 

We next examined the impact of PARP1 depletion on this interaction. PARP1 was depleted using siRNA (Figure S1). As expected, in wild type (WT) conditions PARP1 co-immunoprecipitated IntS3 (Figure 1, Lane 1), while knockdown (KD) of PARP1 resulted little to no co-immunoprecipitated IntS3 (Figure 1, Lane 4). In the confirmatory reciprocal experiment where IntS3 was immobilized to the protein G-beads, very little PARP1 co-immunoprecipitated with IntS3 in PARP1 knockdown cells (assuming that very little is left after knockdown) (Figure 1, Lane 6). The association of PARP1 and IntS3 is specific, as PARP1 did not pull down AF9 (a member of the SEC) or cyclin T1 (CycT1—a member of the P-TEFb complex) in either WT or KD conditions (Figure S2, Lanes 3–4). 

### 3.2. Critical Domains Necessary for Association of PARP1 and IntS3 

#### PARP1 Binds Directly to C-Terminal Domain of IntS3

**Figure 2:**
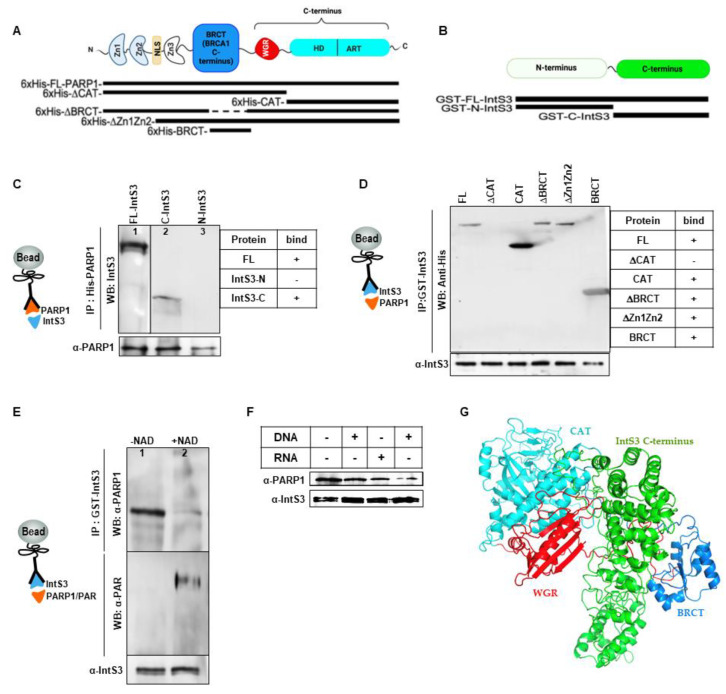
PARP1 and IntS3 form a stable complex in vitro. (**A**) Schematic presentation of PARP1 structural domains and types of PARP1 constructs used in the study. (**B**) IntS3 structural domains and types of proteins used in the study. (**C**) Pull-down of FL-His-PARP1 shows association with both GST-FL-IntS3 (Lane 1) and C-terminal containing IntS3 mutant (C-IntS3, Lane 2) but not IntS3 lacking the C-terminal (N-IntS3, Lane 3). PARP1 was pulled down with His antibody while Western blot analyses with anti-GST antibody show bound IntS3. Anti-PARP1 antibody was used to show the load of PARP1 on the beads. (**D**) GST-tagged FL-IntS3 was incubated with different constructs of PARP1 (His-FL-PARP1, Lane 1), deletion of the catalytic domain (ΔCAT, Lane 2), catalytic domain (CAT, Lane 3), deletion of the BRCT domain mutant (ΔBRCT, Lane 4), deletion of the Z1 and Z2 domains mutant (ΔZ1ΔZ2, Lane 5), and the BRCT-domain-only containing mutant BRCT (Lane 6). Association between IntS3 and these mutants is shown via Western blot analysis with anti-His antibody. Additionally, anti-IntS3 antibody is used to show load of IntS3. (**E**) PARylation of PARP1 does not impact the association of PARP1 and IntS3. PARP1 was incubated without NAD+ (Lane 1) and with NAD+ (Lane 2) to PARylate PARP1. PARylation did not disrupt PARP1-IntS3 binding. (**F**) PARP1-IntS3 binding does not depend on DNA or RNA presence. (**G**) Alphafold [43] prediction of C-terminal of PARP1 binding (BRCT to ART) to C-terminal domain of InSt3.

Having verified that PARP1 and IntS3 associate in vivo, we asked whether this association is direct. We purified and used recombinant His-tagged full-length PARP1 and recombinant GST-tagged full-length IntS3 [41] in reciprocal co-immunoprecipitation studies (Figure S3). In the first case, His-tagged full-length PARP1 (His-FL-PARP1) (Figure 2A) was immobilized through PARP1 antibody on protein-G beads, and then incubated with GST-tagged full-length IntS3 (GST-FL-IntS3) (Figure 2B). After several stringent washes and Western blot analyses of PARP1 and IntS3, we show that FL-PARP1 pulled down FL-IntS3 (Figure 2C, Lane 1). We next asked which domain of IntS3 is important for this binding. We purified deletion mutants of the N and C terminus of IntS3 (Figure S4) and used them in similar co-immunoprecipitation assays. The C-terminal containing mutant (C-IntS3) associated with FL-PARP1, while the mutant lacking this region (N-IntS3) did not (Figure 2C, Lanes 2–3, respectively). These results suggest that the C-terminal of IntS3 is critical for the association with PARP1. 

In the second reciprocal scenario, FL-GST-IntS3 was bound to glutathione beads and then incubated with His-FL-PARP1. Consistent with our observation of a strong in vivo association of PARP1 and IntS3, a direct interaction of purified FL-His-PARP1 with GST-FL-IntS3 was observed (Figure 2D, Lane 1), but not with GST alone (Figure S4). We next asked what domains of PARP1 are important for PARP1 binding to IntS3. Five His-tagged constructs were tested: a construct with deleted CAT domain (ΔCAT); the CAT domain only (CAT); truncation of the zinc-finger DNA binding domain only (ΔZn1ΔZn2); the protein binding domain only (BRCT) and the truncated BRCT domain (ΔBRCT) (Figure 2A). Deletion of the catalytic domain (ΔCAT) resulted in loss of association with the IntS3, while the CAT domain by itself restored the binding (Figure 2D, Lanes 2-3, respectively). These results suggest that the CAT domain of PARP1 is important in its association with IntS3. Supporting this idea, the mutant with truncated DNA binding domain, ΔZ1ΔZ2, did not abrogate the association with IntS3 (Figure 2D, Lane 5). We asked whether other regions of PARP1 might also play a role in this interaction. We first tested the known protein–protein interaction domain, the BRCT domain. Deletion of BRCT (ΔBRCT) did not change the binding of PARP1 and IntS3 (Figure 2D, Lane 4). Interestingly, even the BRCT-domain-only mutant (BRCT) was still able to bind IntS3 (Figure 2D, Lane 6). Our findings, therefore, suggest most likely that more than one domain within the C-terminal region of PARP1 participates in the PARP1–IntS3 association and that PARP1 and IntS3 form a stable complex in vitro.

Since the C-terminal region of PARP1 is important for PARylation as well as for protein–protein interaction, we tested whether PARP1′s PARylation impacts its association with IntS3. We treated His-FL-PARP1 with NAD+, resulting in its autoPARylation (Figure S5) and incubated PARylated PARP1 with GST-FL-IntS3 immobilized on GST beads. Pull-down experiments showed that PARylation on PARP1 did not disrupt the association of PARP1 and IntS3 (Figure 2E). Lastly, we asked whether PARP1-IntS3 binding is dependent on the presence of nucleic acids. We incubated His-FL-PARP1 with either DNA or RNA for 30 min and then performed similar co-immunoprecipitation experiments (Figure 2E). The presence of either DNA (5′-CGT ACG CGG GTT TAA ACG A–3′) or RNA (5′–CGU ACG CGG GUU UAA ACG AG-3′) did not inhibit the formation of the PARP1–IntS3 complex (Figure 2F), suggesting a tight direct interaction between the two proteins. With Alphafold prediction [43], BRCT and WGR domains of PARP1 are close to C-terminal of IntS3 (Figure 2G), suggesting that these domains are critical for its interaction with IntS3. Though deletion of CAT domain resulted in the loss of interaction, it is possible that this is important in the proper conformation of PARP1 needed for a stable association with IntS3.

### 3.3. IntS3 Is Not PARylated by PARP1

Since the activation of NELF requires PARylation by PARP1, we speculated that PARP1 might PARylate IntS3 for activation. IntS3 was immunoprecipitated from S2 nuclear extracts and subjected to PAR Western blot analyses (Figure S5A). No PARylation of IntS3 was observed. We then performed a reciprocal experiment, where PAR was immunoprecipitated with PAR antibody and Western blot analysis was performed with an IntS3 antibody. We again failed to detect PARylation on IntS3, though we showed PARylation of PARP 1 (Lane 2). We posited that this failure in observing PARylation on IntS3 might be due to low levels of PARylation in the cells. We therefore instigated PARP1 PARylation by adding NAD+ to cells and in another scenario, cells were heat shocked for 15 min at 37 °C (Figure S5B, Lanes 2–3). Even in these conditions, while PARP1 was PARylated, we failed to observe the PARylation of IntS3. These studies are in line with Vivelo et al., 2017 [44], who showed that IntS3 is mono (ADP)-ribosylated, not PARylated. To further confirm this finding, we PARylated recombinant PARP1 by incubating PARP1 with DNA and NAD+, performed PAR IP and Western blotted for IntS3 (Figure S5B). In this analysis, although we observed the interaction of IntS3 with PAR, we did not observe PARylation on IntS3. These results of IntS3 interacting with PAR suggest that in vitro IntS3 might be interacting with PARylated PARP1. In follow-up experiments, we treated the cocktail of PARylated PARP1, NAD+, and DNA with PJ34, to inhibit PARP1 PARylation. PAR pull-down again showed interaction with IntS3. We assume that the interaction of PAR and IntS3 in this case is due to PARylated PARP1 and not PARylation of IntS3. In summary our in vivo and in vitro data support findings from other studies that IntS3 is not PARylated [45].

### 3.4. PARP1 Modulates the Chromatin Association of Elongation Factors

**Figure 3:**
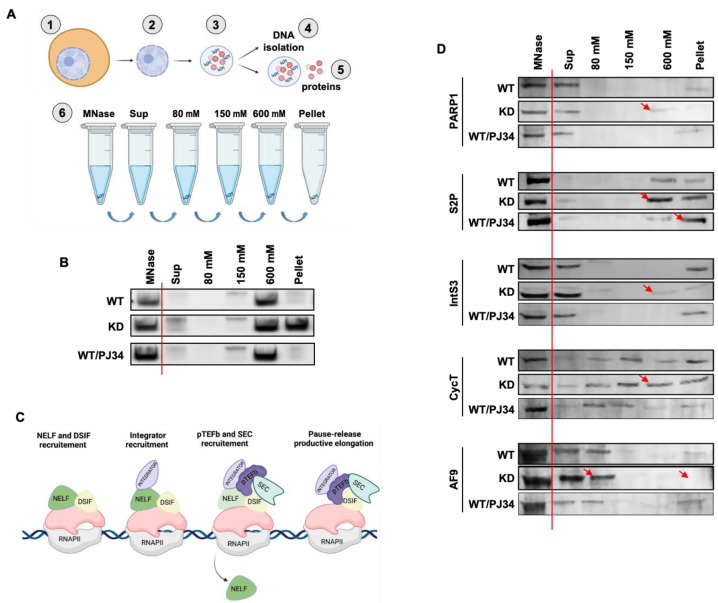
PARP1 modulates the subcellular localization of key elongation factors. Chromatin salt fractionation of PARP1, RNAPII, and elongation factors in WT-, PARP-KD-, and PJ34-treated cells. (**A**) Schematic representation of nuclear fractionation protocol. Chromatin from ~5 × 10^7^ cells was digested to mono-nucleosome length using MNase and the digested chromatin subjected to salt fractionation with increasing salt concentrations. DNA isolated from each step was used to confirm validity of protocol. (**B**) DNA released under the different salt concentrations was analyzed on a 4% NuSieve™ agarose gel. (**C**) Known model of the association of elongation complexes during RNAPII elongation in the gene body. We then tested the effect of PARP1 on the integrator and SEC complexes. (**D**) Western blot of different salt fractionation steps with antibody against PARP1, RNAPII elongating (S2P), IntS3 and several chromatin-associated elongation factors (Integrator: IntS3), p-TEFb (CycT) and super elongation complex (AF9). Arrows indicate differences observed in protein shifts.

To understand the influence of PARP1 on transcription elongation factors, we digested chromatin from WT-, KD-, and PJ34-treated cells with micrococcal nuclease (MNase) and subjected the samples to salt fractionation [46]. The samples were split in two for DNA isolation and Western blot analyses (Figure 3A). Resultant DNA samples were quantified by Nano-Drop and run on a 4% NuSieve™ agarose gel. A salt-dependent differential release of chromatin from cells was observed in WT, KD, and PJ34 conditions. In WT cells, the major chromatin band was in the 600 mM salt fraction with almost nothing left in the pellet heterochromatin fraction. Interestingly, in PARP1-depleted cells (KD), there was a strong band in the pellet fraction, indicating a denser chromatin state under PARP1 depletion. PARylation inhibition, on the other hand, did not change chromatin states when compared to WT condition (Figure 3B). 

We next analyzed whether PARP1 impacts the sequential recruitment/assembly and chromatin association of key elongation factors. At the promoter, RNAPII elongation starts with the recruitment of DSIF-NELF (DRB sensitivity-inducing factor and negative elongation factor)**,** followed by the recruitment of the Integrator complex, then P-TEFb and finally the SEC (Figure 3C). We used Western blot analysis to determine the chromatin association of PARP1, RNAPII, and elongation factors of the Integrator, P-TEFb and SEC complexes (Figure 3C). 

In WT cells, PARP1 was found mainly in the supernatant fraction after MNase digestion (total chromatin) and in the pellet (dense heterochromatinized) fraction. In KD cells, PARP1 redistributed to the 600 mM salt fraction rather than to the pellets (red arrow—Figure 3D), indicating the presence of PARP1 in more soluble chromatin compared to WT conditions. In PJ34-treated cells, there was no change to the PARP1-chromatin association when compared to conditions in WT cells. We also analyzed the distribution of the elongating RNAPII by measuring the occupancy of Ser2 phosphorylated RNAPII. We found that in WT conditions Ser2 RNAPII was equally distributed in the 600 mM and pellet fractions (both heterochromatin regions), while in PARP1 KD conditions, Ser2 RNAPII was found mainly in the 600 mM fraction (red arrow) compared to the pellet fraction. Interestingly, when PARylation was inhibited, Ser2 RNAPII associated mainly in the most heterochromatinized pellet fraction compared to the 600 mM fraction. This finding of RNAII in the dense pellet fraction is in line with previous studies showing RNAPII associating with the most insoluble chromatin fractions [47]. In PARP1 WT S2 Drosophila cells, IntS3 associates mainly with the very loose chromatin found in the supernatant fraction while a smaller amount was found in the pellet fraction. In KD conditions, IntS3 is mainly found in the supernatant fraction and is redistributed in the 80 mM, 600 mM, and pellet fractions (Figure 3D). This seems to suggest a looser binding of IntS3 with chromatin in the absence of PARP1. When cells were treated with PARylation inhibitor, there was no significant change in IntS3 association with chromatin when compared to WT conditions. In general, IntS3 distribution and redistribution corresponds with PARP1′s. We next used CycT1 and AF9 antibodies to track P-TEFb and SEC complexes, respectively. In WT conditions, CycT1 is loosely bound to chromatin, as it is eluted in all fractions, with maximum elution occurring in the 150 mM (loose chromatin) and pellet fractions. When PARP1 was knocked down, the maximum band intensity was equally distributed between the 150 mM, 600 mM, and pellets fractions (red arrow in Figure 3D). There was no visible difference between WT and PJ34 conditions. For AF9, the most visible bands were in the supernatant and 80 mM fractions, with a very weak band in the pellet fraction. These results suggest that AF9 is mainly associated with loose active chromatin in both WT- and PJ34-treated cells. In PARP1 KD cells, a stronger band was observed in 80 mM, but no band was seen in the pellet fraction (red arrows—Figure 3D). In summary, these data show that in the absence of PARP1, there is a redistribution of the association of elongation factors and RNAPII with different chromatin fractions. These factors seem to be associated more often with more soluble chromatin, which could suggest increased gene expression. To determine if this is the case, we subsequently analyzed their location and occupancy at two PARP1 target genes *AKAP200* and *CAPER*, which we have extensively studied [31,48].

### 3.5. PARP1 and PARylation Impact Localization and Occupancy of Elongation Factors along the Gene Body

**Figure 4:**
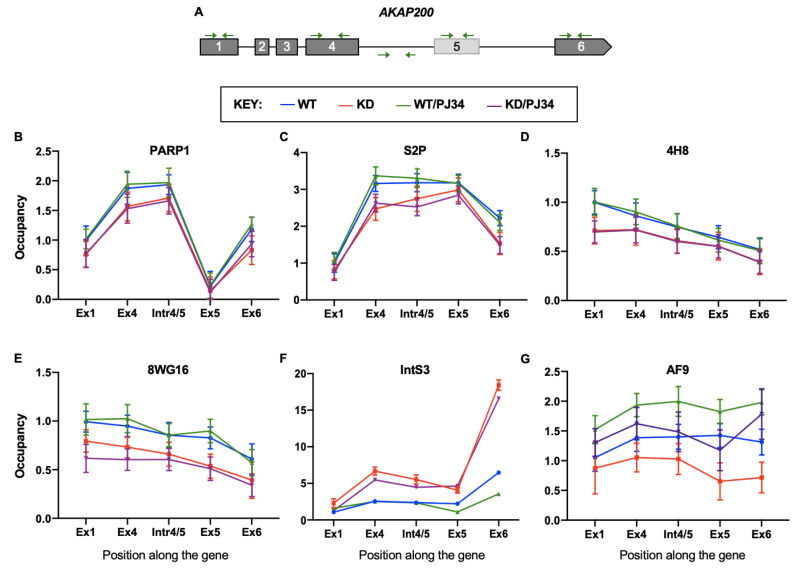
PARP1 and PARylation differentially impact localization/occupancy of elongation factors along the gene body of the *AKAP200* gene. (**A**) Cartoon showing the location of primers used along the gene body of the *AKAP200* gene. qPCR analyses of the occupancies of (**B**) PARP1, (**C**) elongating form of RNAPII (S2P), (**D**) Initiating form of RNAPII (4H8), (**E**) Initiating form, which is sometimes used for total RNAPII (8WG16). (**F**) Integrator subunit 3 (IntS3) and (**G**) AF9 (a member of the super elongation complex), along the *AKAP200* gene body. All experiments were performed in triplicate, and results are presented as mean ± SD (the differences between treatment conditions observed, were statistically significant with a *p* value < 0.05). Statistical significance was tested by Student’s t-test method.

During transcription, RNAPII associates with elongation factors; we therefore asked whether PARP1 influences the recruitment/distribution of these elongation factors along the gene body. We measured the occupancy of PARP1, RNAPII, Integrator, SEC, and P-TEFb complexes along the gene body of two PARP1 target genes. Our experimental design included four different conditions of PARP1/PARylation in S2 *Drosophila* cells: wild type cells (WT, blue line), PARP1 knockdown cells (KD, red line), wild-type cells treated with the PARylation inhibitor PJ34 (WT/PJ34, green line), and PARP1 knockdown cells treated with PJ34 (KD/PJ34, purple line). First, we analyzed the occupancy of PARP1 along the gene body of two PARP1 target genes—*AKAP200* and *CAPER*. We created primer sets for different gene body locations: (1) first exon, (2) the immediately preceding constitutive exon, (3) the intervening intron, (4) the alternative exon, and (5) the last exon of these genes, as shown in Figure 4A for *AKAP200* and Figure S6A for *CAPER*. 

In both WT and WT-PJ34, PARP1 occupancy was high in regions of the gene body just before alternative exon 5, with the highest enrichment in regions just upstream of the alternative exon (i.e., exon 4 and the intron preceding alternative exon 5, as shown on Figure 4B for *AKAP200*; exon 3 and the intron before alternative exon 4, as shown on Figure S6B for *CAPER*). As expected, PARP1’s occupancy along the gene body decreased in KD cells. Inhibition of PARylation did not have any impact on PARP1 occupancy, as similar results were observed in WT vs. WT/PJ34 cells (Figure 4B, blue line vs. green line, respectively) and in KD vs. KD/PJ34 cells (Figure 4B, red line vs. purple line, respectively). Occupancy of PARP1 at the *CAPER* gene mimicked that of the *AKAP200* gene (see Figure S3B). 

Next, we asked whether the observed changes in PARP1 occupancy correlate with changes in the occupancy of different RNAPII forms. Using a Ser2P antibody (which recognizes the elongating form of RNAPII), we showed that elongating RNAPII occupancy correlated with PARP1 occupancy along the gene body (Figure 4C). Interestingly, in WT conditions at the alternative exon, Ser2P occupancy was high. However, when we compared the ratio of the levels of Ser2P in WT vs. KD conditions at the different genic regions, RNAPII was reduced at exon 4 but more enriched at intron 4/5 and exon 5 (Figure 4C). Again, we made a similar observation where Ser2 RNAPII is reduced at exon 3 but increased at intron 3/4 and alternative exon 4 in the *CAPER* gene in WT vs. KD conditions (Figure S6C). These results suggest that the roadblock mediated by PARP1 is alleviated after PARP1 knockdown, allowing RNAPII to move further into the gene body. Next, we analyzed whether this effect by PARP1 is specific only for elongating RNAPII occupancy. As expected, the initiating Ser5P RNAPII (4H8) (Figure 4D and Figure S6D) and the hypo-phosphorylated pre-initiating RNAPII (8WG16) (Figure 4E and Figure S6E) were decreased in the gene bodies of both *AKAP200* and *CAPER,* respectively. However, KD of PARP1 significantly further decreased the occupancy of these two RNAPII forms (*p*-value < 0.05). Inhibition of PARylation had no effect on the genomic occupancy of these RNAPII forms (see WT vs. WT/PJ34 and KD vs. KD/PJ34), suggesting a direct effect of PARP1 on RNAPII occupancy. 

Next, we measured the occupancy of IntS3 in *AKAP200* (Figure 4F) and *CAPER* (Figure S6F). Changes in the distribution of IntS3 in WT-, PARP1-KD-, and PJ34-treated cells were more drastic. In KD cells, the total amount of IntS3 was higher compared to WT cells. These data are in accordance with our previous finding of the increased expression of IntS3 in PARP1 KD cells [31]. One possible explanation for the increased occupancy of IntS3 along the target genes after PARP1 KD is that the overall expression of IntS3 is increased after PARP1 KD, and not necessarily due to increased recruitment/release of the roadblock to RNAPII elongation complex in PARP1 KD. We ruled this out by measuring the occupancy of IntS3 along a PARP1 non-target gene (*Actin 5C*) in all conditions. We show that although IntS3 expression was generally high in PARP1 KD cells, there was no change in the distribution and occupancy of IntS3 along the *Actin 5C* gene (Figure S7). These results suggest a direct effect of PARP1 on IntS3 occupancy and distribution on PARP1 target genes. An interesting observation on the distribution of IntS3 is the high occupancy towards the end of the genes, supporting the involvement of IntS3 in the transcription termination process [49,50].

Finally, we measured the occupancy of AF9 (a member of the SEC complex) along the gene body. In WT conditions, AF9 was equally distributed along the gene bodies of *AKAP200* (Figure 4G, blue line) and *CAPER* (Figure S6G, blue line). In KD cells, there was a slight decrease in the overall occupancy with an even greater decrease at alternative exon 4 for *AKAP200* (Figure 4G, red line) and exon 5 for *CAPER* (Figure S6G, red line). Interestingly, when PARylation was inhibited in WT cells (Figure 4G, green line) and KD cells (Figure S6G, purple line), AF9 occupancy levels were elevated throughout the gene body. Furthermore, the relatively slight decrease seen at the alternative exons in KD cells was even more pronounced in cells with PARylation inhibition. Our results, therefore, show that while with some elongation factors such as IntS3, the presence of the PARP1 protein rather than its PARylation effect is important; in others, such as with AF9, the PARylation effect is greater. It is possible that AF9 is PARylated by PARP1 impacting its regulatory functions in transcriptional elongation, but that will need to be tested in future studies.

## 4. Discussion

RNAPII elongation initiates at the promoter, progresses through the gene body and terminates at the end of the transcript. These steps are tightly coordinated and regulated for proper gene expression. A long-held paradigm is that the pausing of the polymerase at the promoter is critical for transcription initiation. However, recent evidence shows that once the polymerase transitions into productive elongation, it faces other barriers. Thus, transcription elongation is now known to be a rate-limiting step, governing both splicing decisions and gene expression [51,52]. How these barriers are relieved and the factors regulating the release are still being investigated. 

Pause–release of RNAPII elongation from both the promoter and within gene bodies is implicated in splicing regulation. Although PARP1’s role in the RNAPII pause–release at promoters is recognized [27,28,29,31,32], very little is known about its role in gene body pause–release. We showed that a PARP1-mediated chromatin structure slows the rate of RNAPII elongation [31]. One possibility is that PARP1-bound chromatin regulates the assembly/stability of elongation factors at paused sites within the gene body. A kinetic understanding of PARP1-mediated recruitment/assembly of elongation factors may be key in delineating its role in RNAPII elongation and splicing. From transcription initiation, RNAPII elongation starts with the sequential recruitment of the DSIF-NELF, followed by the Integrator complex. For pause–release, this DSIF-NELF-Integrator complex then recruits P-TEFb, followed by the SEC [34,35,36], forming the central elongation scaffold [37]. Interestingly, for paused RNAPII to move into productive elongation NELF-E is PARylated by PARP1, resulting in a CDK9/P-TEFb-mediated NELF-E phosphorylation [53]. 

In this study, we set out to understand whether PARP1 aids in the recruitment/assembly of elongation factors once it encounters a PARP1-mediated chromatin structure. We show that: (1) IntS3 is part of the PARP1-interactome [29]; (2) PARP1 co-immunoprecipitates with IntS3 in vivo (Figure 1); (3) in vitro studies using recombinant proteins show direct binding between these two proteins (Figure 2); and (4) using salt chromatin fractionation studies, we show that depletion of PARP1 KD, not PARylation, caused differential chromatin association of the elongation of AF9 and CycT1 (Figure 3). Based on these findings, our working hypothesis is that PARP1 binds to IntS3 in the recruitment/assembly of the SEC and/or the P-TEFb complex for proper pause–release. 

Our data show that PARP1 impacts IntS3 occupancy along gene bodies of PARP1 target genes, *AKAP200* and *CAPER*. Knockdown of PARP1, not PARylation, resulted in decreased PARP1 occupancy along the gene (Figure 4B), with a coincident increase in occupancy of IntS3 (Figure 4F). We then asked what happens to the SEC components because of PARP1. In our previous studies, we had observed decreased expression of AFF4 and AF9 (both SEC components) in PARP1 KD cells [29]. Interestingly, both PARP1 [54] and ELL [55], another SEC member [37], have been shown to regulate poised RNAPII transcription at the heat-shock promoter through PARP1’s stabilization of ELL to form a stable elongation complex [56]. Building on this finding, we analyzed whether PARP1/PARylation influences SEC recruitment. We showed that AF9 binding, and occupancy correspond with PARP1 binding, suggesting that PARP1 occupancy may also be influencing the occupancy of AF9. It is possible that because PARP1 is affecting the upstream complex (Integrator complex), the downstream complexes might not be assembled correctly. Such changes will therefore have an impact on the fidelity of transcription elongation and subsequent gene expression. In most cases, while the presence of PARP1 had an effect, PARylation had more or less little impact on PARP1 regulation of the occupancy of these elongation factors. 

Deletion (depletion) of IntS3 results in the failure of RNAPII to escape pausing [57]. In WT cells with normal PARP1 levels, we observed a basal level of IntS3 expression. After PARP1 is depleted, the global expression of IntS3 and its occupancy along PARP1-target genes was enhanced, suggesting a relief in pausing and more RNAPII elongation. One would expect that the enhanced expression is global, affecting the occupancy along all genes. However, we posit that this is PARP1-mediated, as the enhanced occupancy of IntS3 was not observed along the *Actin 5C* gene, a PARP1-nontarget gene. These findings are consistent with other studies showing that IntS3 and IntS11 regulate RNAPII occupancy and processivity at NELF-target genes [49]. Since PARP1 associates with NELF [53], and IntS3 is known to interact with NELF and Spt5 in the absence of other IntSCom subunits and RNAPII, it is possible that PARP1 is part of the NELF–Spt5–IntS3 scaffolding complex that is critical for relief of RNAPII pausing. In some pause–release instances at the promoter, PARP1 PARylates NELF; we asked whether PARP1 also PARylates IntS3 for activity. We showed that IntS3 is not PARylated both in vivo and in vitro. These data are in line with previous global studies showing that IntS3 is PARylated by PARP2 [44]. While our studies show no PARylation of IntS3 by PARP1, it still possible that other members of the complex are PARylated, thus causing dissociation of key components critical in RNAPII pause/release. Future studies will be needed to determine if any other member of the complex is PARylated for activity or if PARP1 functions only as a scaffolding hub. 

In summary, determining the factors regulating the rate-limiting steps in transcriptional control is of fundamental importance for understanding the mechanisms that govern eukaryotic transcription. While studies in unicellular organisms have pointed to “initiation” as the rate-limiting step in transcription, a large body of work in metazoans indicates that the transition to productive transcriptional elongation may also constitute a critical step. Thus, this study focuses on PARP1′s role in the rate of nascent RNA growth and how its association with elongation factors impacts this role. Thus, by associating with elongating RNAPII and elongation factors, PARP1 may be contributing to transcriptional fidelity through scaffolding/recruiting of these pause–release factors for proper gene regulation. 

## Data Availability

All data needed to evaluate the conclusions in the paper are present in the paper and the Supplementary Materials. Additional data related to this paper may be requested from the authors.

## Supplementary Materials

The following supporting information can be downloaded at: https://www.mdpi.com/article/10.3390/cells11203202/s1, Figure S1: Knockdown of PARP1 in S2 Drosophila cells. Figure S2: PARP1 associates IntS3 in S2 Drosophila cells but PARP1 does not associate with the other elongation factors (CycT1 and AF9); Figure S3: Purification of His-tagged PARP1 constructs; Figure S4: Purification of IntS3 constructs; Figure S5: IntS3 is not PARylated both in vivo and in vitro; Figure S6: PARP1 and PARylation differentially impact localization/occupancy of elongation factors along the gene body of the *CAPER gene*; Figure S7: IntS3 occupancy and location is not affected on *Actin 5C* gene body, a PARP1 non-target gene; Table S1: Primers used in the study.
