# Supplementary material for: PARP1′s Involvement in RNA Polymerase II Elongation: Pausing and Releasing Regulation through the Integrator and Super Elongation Complex
Source: Cells. 2022 Oct 12;11(20):3202. doi: 10.3390/cells11203202 (PMC9600911; doi:10.3390/cells11203202)
Supplement: Supplementary file 1 [file cells-11-03202-s001.zip › cells-1930251-supplementary.pdf]

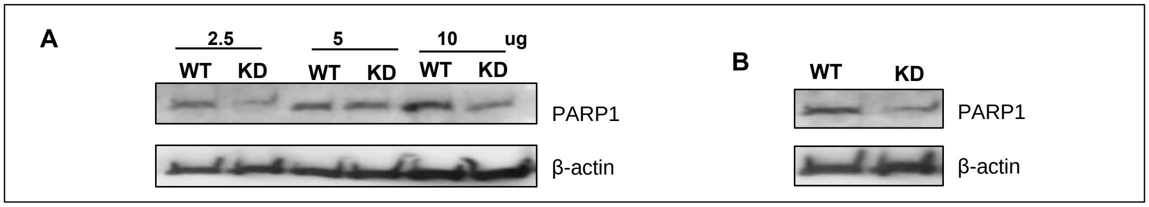

Figure S1. Knockdown of PARP1 in S2 Drosophila cells. **Supplemental Figure S1**. Knockdown of PARP1 in S2 Drosophila cells. B-actin is used as loading control. **A.** A serial dilution of the total proteins is done to show the degree of PARP1 knockdown. **B.** Shown is the 2.5 ug of total protein loaded on the gel from A, showing the knockdown of PARP1.

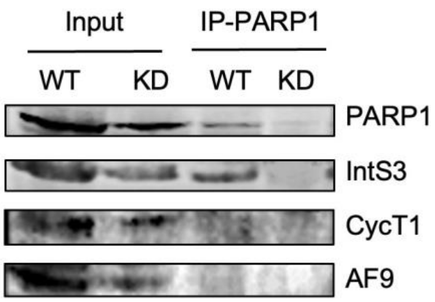

Figure S2. PARP1 associates IntS3 in S2 Drosophila cells but PARP1 does not associate with the other elongation factors (CycT1 and AF9). Samples were blotted with antibodies against key members of the elongation complexes (CycT1 for pTEFb and AF9 for SEC).

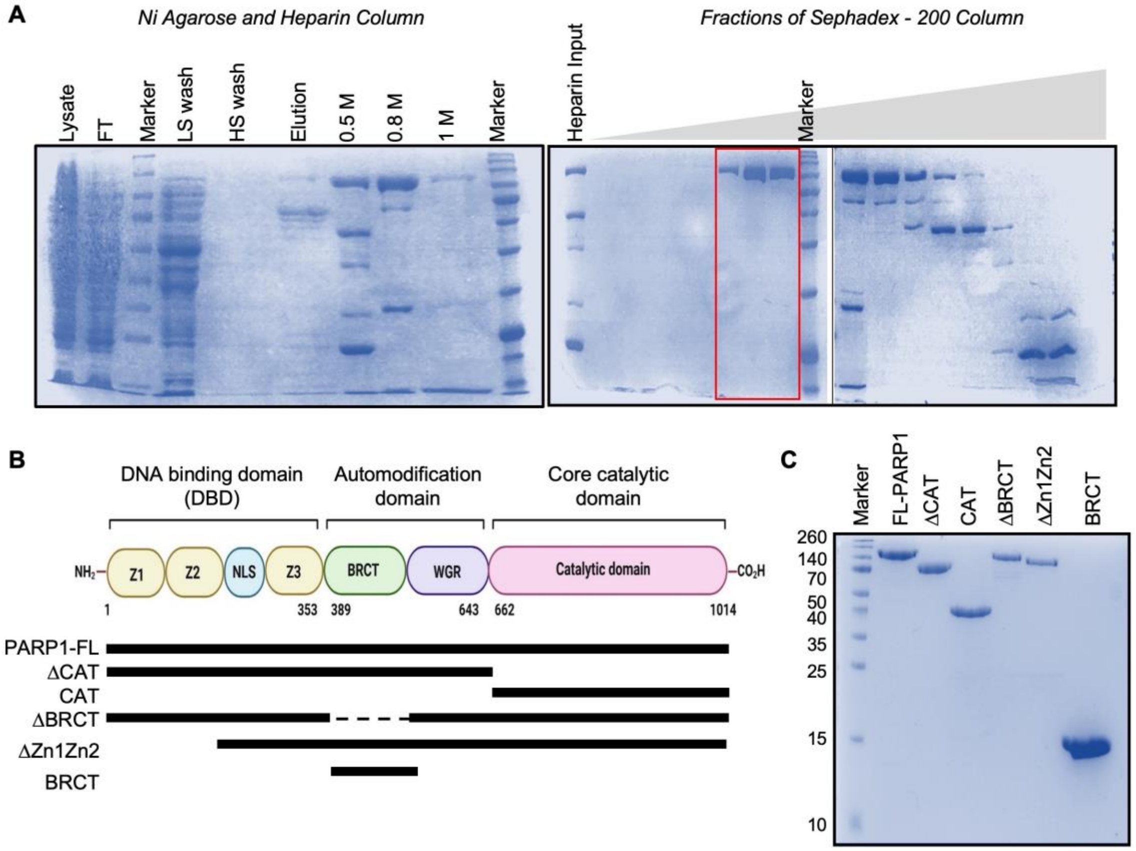

Figure S3: Purification of His-tagged PARP1 constructs **A**. SDS–PAGE analysis of His-tagged FL-PARP1 protein eluted from Nickel and Heparin columns. Marker - The positions of protein markers are shown. **B.** Different fractions of the recombinant GST-FL-PARP1 protein eluted from a Sephadex-200 column. Clean and purified protein fractions (red box) were collected and used for studies. **C.** Commassie staining of the various purified PARP1 proteins used in these studies.

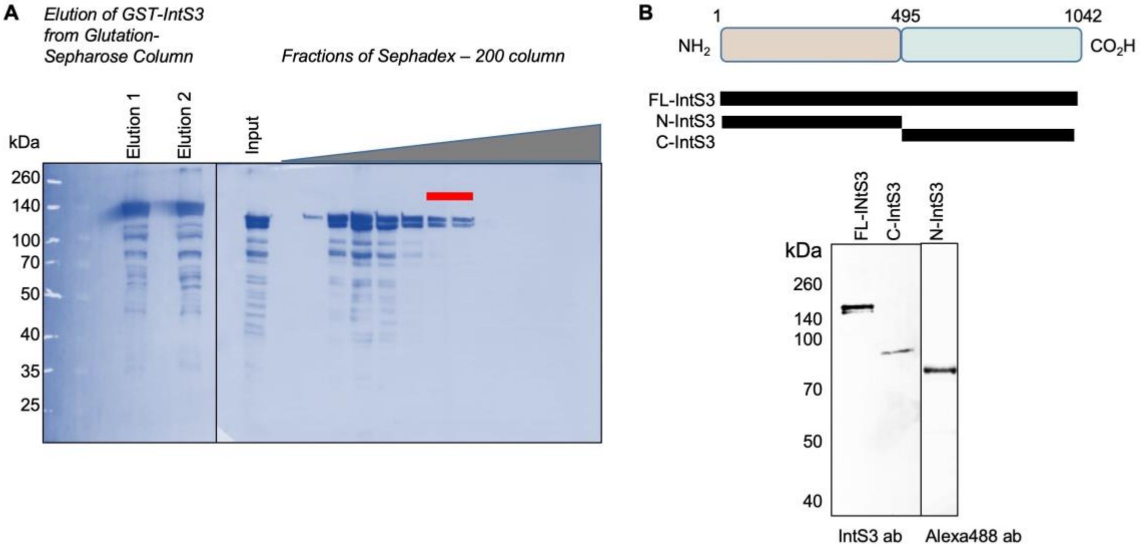

Figure S4. Purification of IntS3 constructs: **A.** SDS–PAGE analysis of GST-FL-INTS3 protein eluted from glutathione resin digested with PreScission protease. Further purification was carried out on a Sephadex-200 column. Clean protein fractions (red line) were used for studies. The positions of protein markers are shown. **B.** Western blot analysis was performed to analyze the purified proteins using an anti-IntS3 antibody (which recognizes both FL-IntS3 and C-IntS3 but not N-IntS3). To probe for N-IntS3, Alexa 488 antibody, which probes for GST was used.

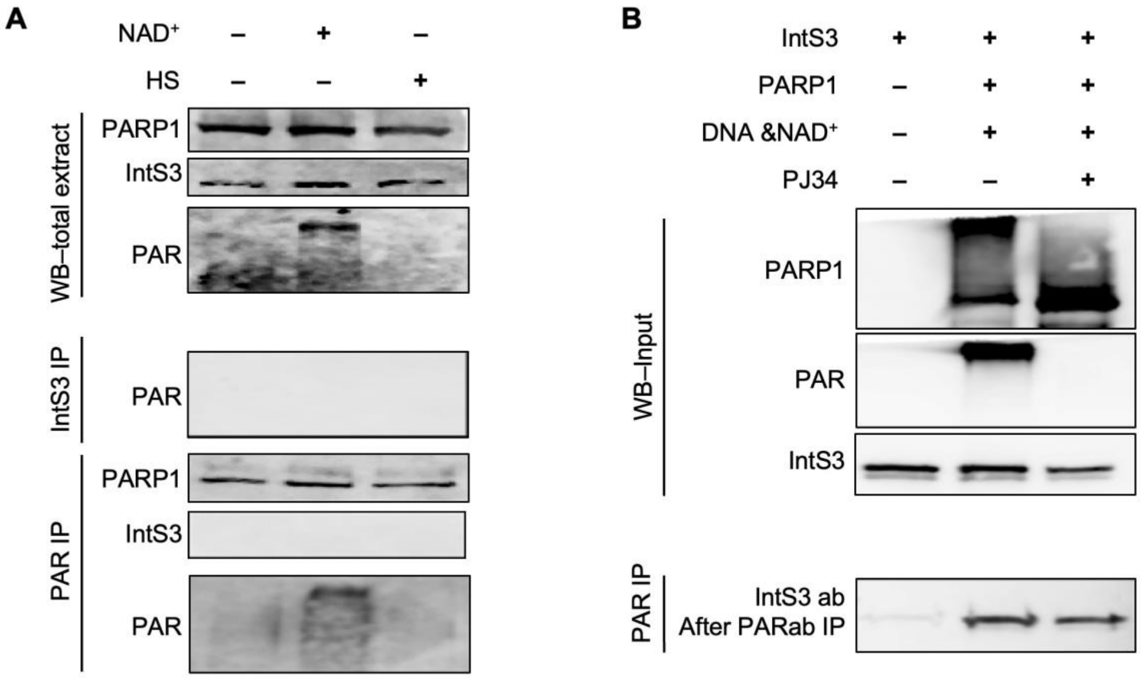

Figure S5. IntS3 is not PARylated both in vivo and in vitro. Nuclear extracts from S2 Drosophila cells were subjected to immunoprecipitation under conditions in which PARP1 can be PARylated (adding of NAD+ to cells or HS conditions. **A.** These extracts were subjected to an immunoprecipitation assay using IntS3 antibody. **B.** Recombinant PARP1 was incubated with either vehicle, NAD+ or PJ34 as indicated. Western blot assays were used to detect PARP1, PAR and IntS3 (input). Additionally, PAR was IPped, followed by western blot analysis of IntS3. We assume the latter association seen is due to PARP1 binding directly to IntS3 and not due to IntS3 PARylated.

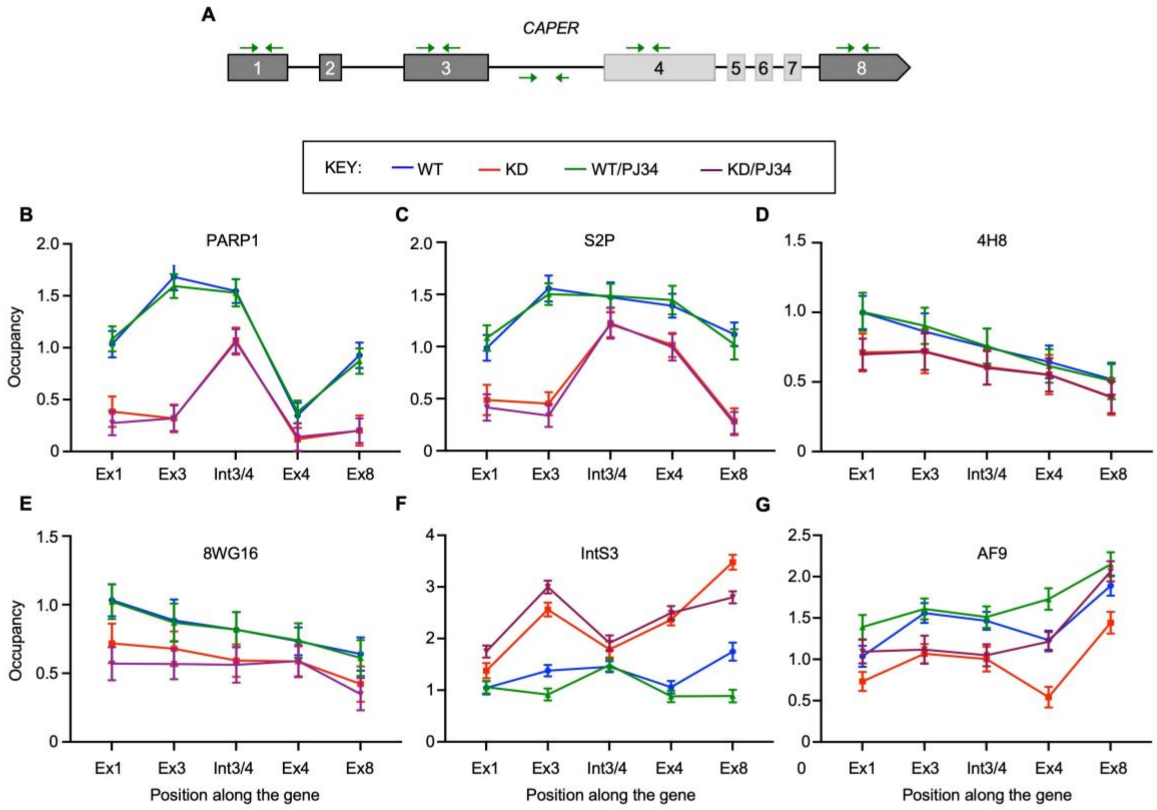

Figure S6. PARP1 and PARylation differentially impact localization/occupancy of elongation factors along the gene body of the *CAPER gene*. **A**. Cartoon showing the location of primers used along the gene body of the *AKAP200* gene. qPCR analyses of the occupancies of **B**. PARP1, **C.** elongating form of RNAPII (S2P), **D.** Initiating form of RNAPII (4H8), **E**. Initiating form, which sometimes used for total RNAPII (8WG16). **F.** Integrator subunit 3 (IntS3) and **G.** AF9 (a member of the super elongation complex), along the *CAPER* gene body. All experiments were performed in triplicate, and results are presented as mean ± SD (p value < 0.05). Statistical significance was tested by Student's t test method.

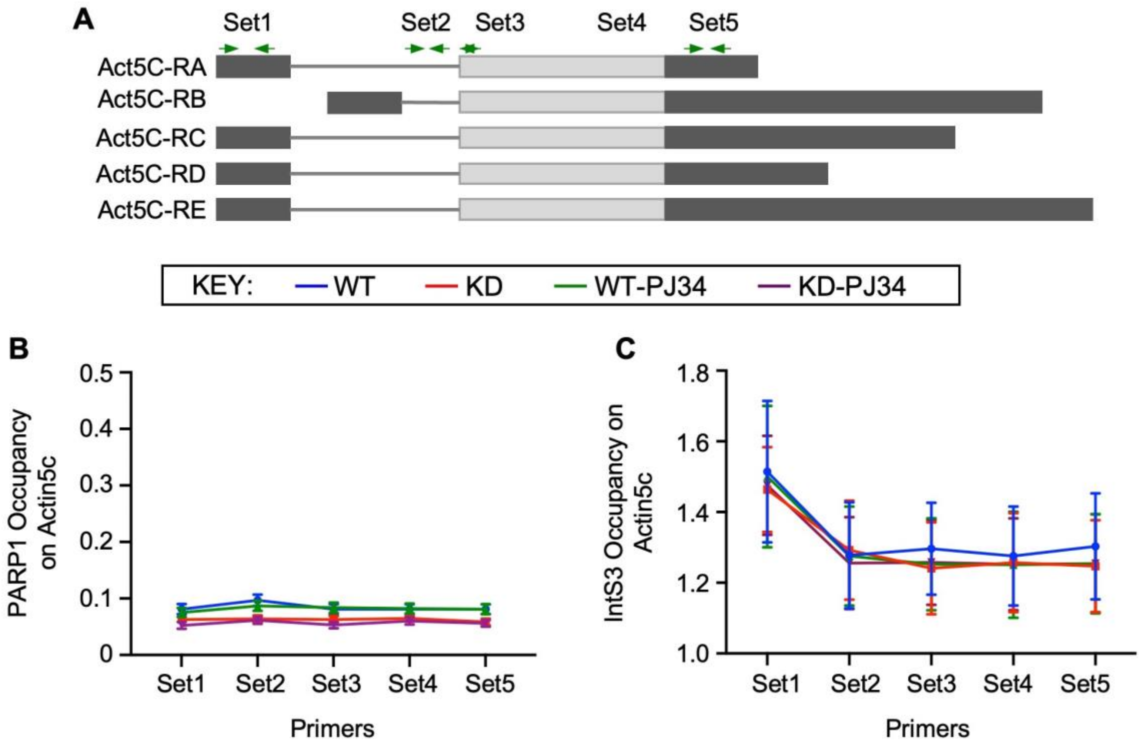

Figure S7. IntS3 occupancy and location is not affected on *Actin 5C* gene body, a PARP1 non-target gene. **A**. Cartoon showing the location of primers used along the gene body of the *Actin 5C* gene. qPCR analyses of the occupancies of **B**. PARP1, **C.** IntS3 along the *Actin 5C* gene body. All experiments were performed in triplicate, and results are presented as mean ± SD (p value < 0.05). Statistical significance was tested by Student's t-test method.

Table S1. Primers used in the study

Gene	#	Primer name	Direction	Primer sequence (5'-3')
PARP1	1	PARP1/F	Forward	TCGACGTGTCGTGGATGTGAACAA
PARP1	2	PARP1/R	Reverse	ACAAAGGTTGGCCTCCGTACTTCA
Actin	3	Actin5C/F	Forward	TCGCGATTTGACCGACTACCTGAT
Actin	4	Actin5C/R	Reverse	TTGATGTCACGGACGATTTCACGC
Akap	5	AkEx1/F	Forward	ACATCCTAACGCGACGTAAATA
Akap	6	AkEx1/R	Reverse	CTGCTTTCCGTTTCGGTTTC
Akap	7	AkEx4/F	Forward	CTGCTGCTGGTGAGGATATAA
Akap	8	AkEx4/R	Reverse	GTCCTTCTTGCCAAAGGAAATG
Akap	9	AkEx5/F	Forward	AGTTGAAGCCAAGTCCGTAG
Akap	10	AkEx5/R	Reverse	TCCACAATAACGGACTCGAAC
Akap	11	AkEx6/F	Forward	GATCTCGCCAAGGATCTGAA
Akap	12	AkEx6/R	Reverse	GAGTAGGATTATTCGCATGTAACG
Akap	13	AkInt4/5/F	Forward	AGAGTTAGTTCAGACCCAAACA
Akap	14	AkInt4/5/R	Reverse	GGGTCACTGTTTCAATCACTTC
Caper	13	CapEx1/F	Forward	TCGATAAGTTCTGTGACAACCC
Caper	15	CapEx1/R	Reverse	CGCACAGCGGAATTCGTA
Caper	16	CapEx3/F	Forward	CCCGAATTGCAGCGAAGTA
Caper	17	CapEx3/R	Reverse	TTATCCGGCCTTTGGGAAC
Caper	18	CapEx4/F	Forward	TGTCTTTGCAGAGAATCGAATAAG
Caper	19	CapEx4/R	Reverse	CAGCTTCTGGCACATTCAAA
Caper	20	CapEx8/F	Forward	GTGGACACGATGACGACTAC
Caper	21	CapEx8/R	Reverse	GGGATCGTTTGCTTTGCTT
Caper	22	CapInt3/4/F	Forward	CACACTAGACTGCGTTCATACA
Caper	23	CapInt3/4/R	Reverse	GTATCAAATGCCGCGTAGGA
Intergene	24	InG/F	Forward	TTAAGGCATTTTAGTCATCTTAATTGTATG
Intergene	25	InG/R	Reverse	GGCTTCGGGTCTAGTTATAGTG
